# Ultrasound-Guided Continuous Radiofrequency Ablation Of Painful Residual Limb Neuroma In Individuals With Limb Amputation-A Retrospective Case Series

**DOI:** 10.33137/cpoj.v2i1.33061

**Published:** 2019-11-03

**Authors:** S. Guo, R. Mansour, D. Henderson Slater

**Affiliations:** 1 Oxford Centre for Enablement, Nuffield Orthopaedic Centre, Oxford, UK.; 2 Department of Radiology, Nuffield Orthopaedic Centre, Oxford, UK.

**Keywords:** Prosthetics, rehabilitation, amputations, neuroma, pain, radiofrequency ablation

## Abstract

**BACKGROUND:**

Residual limb neuromas are a significant cause of post-amputation pain. There is little knowledge concerning ultrasound-guided (US) radiofrequency ablation (RFA) as treatment.

**OBJECTIVE:**

To investigate US-guided RFA for neuroma associated pain in individuals with limb amputation.

**METHODOLOGY:**

The notes of nine consecutive patients were retrospectively reviewed. Information obtained included neuroma size and nerve, RFA duration/temperature, pain scores, analgesic requirements and ease/comfort of prosthetic use. Eight patients had lower-limb amputations and one had a trans-radial amputation. All except one, underwent diagnostic US-guided steroid injection to confirm the neuroma as the source of pain, prior to RFA.

**RESULTS:**

Six patients reported significant reduction in pain scores (defined as at least 50% reduction) and an improvement in comfort/ease of wearing their prosthetic limb, with no adverse effects. Three of these six patients also reported a reduction in analgesic requirements. Of the three remaining patients – one had a large sciatic nerve neuroma that was eventually surgically excised, another had confounding pain from an adjacent bony spur, whilst the third patient did not receive a routine diagnostic steroid injection prior to RFA.

**CONCLUSIONS:**

Our findings suggest that US-guided RFA is safe and effective for small to medium-sized residual limb neuroma associated pain in individuals with limb amputation. It can reduce pain and analgesic requirements, improve comfort/ease of wearing the prosthesis and potentially avoid surgical excision. We recommend patients should undergo a diagnostic steroid injection prior to RFA to confirm that the neuroma is the source of pain.

## INTRODUCTION

Post-amputation pain is very common after limb amputation, and residual limb neuroma is a significant cause of this, which can include both pain around the residual limb and phantom pain. However, it is also recognised that many individuals with limb amputation have neuromas that are not apparently causing pain.^1^ Neuromas are commonly seen as incidental findings on scans carried out for other clinical reasons, and the precise degree of correlation between the presence of a neuroma and phantom pain due to the neuroma is not known.^2^

Residual limb neuroma-associated pain can be difficult to treat and is often managed using an interdisciplinary approach with a combination of neuropathic analgesia, physiotherapy, adjustments to the prosthesis, prosthetic counselling and eventual surgical excision.^3^

Neuroma-associated pain could be due to residual limb pain or phantom pain or a combination of both. Residual limb pain (also known as stump pain) has been described as a sharp, burning, ‘electric shock’-like pain which the patient often attributes to an incision site around the residual limb or perceives the pain originating deep in the residual limb.^4^ Its incidence has been reported in up to 74% of individuals with limb amputation.^5^ This is different from phantom pain, which has been described as a painful or unpleasant sensation in the distribution of severed limb after amputation. It can be classified as either neuropathic-like (sharp, shooting, electric shock type pain) or nociceptive-like (dull, squeezing, and cramping) or a combination of both. It has been reported in 85% of patients with limb amputation. ^4^

Although the mechanism of interaction is not fully understood, residual limb pain and phantom pain often coexist post-amputation and Montoya et al have reported a significant correlation between the severities of these two types of pain in individuals with limb amputation.^6^

There is a paucity of studies in the literature concerning ultrasound-guided (US) radiofrequency ablation (RFA) as a treatment for residual limb neuroma pain in terms of its effectiveness in reducing pain and analgesic requirements, improving comfort when wearing prosthetic limbs and reducing the need for surgical intervention

This study aims to investigate the effects of US-guided RFA as a treatment for residual limb neuroma- associated pain. After retrospectively reviewing the patient case notes, we retrieved data on the safety, efficacy, side effects, and complications of US-guided RFA in the treatment of painful residual limb neuroma.

## METHODOLOGY

### RETROSPECTIVE REVIEW

The clinical notes and imaging of nine consecutive patients who underwent RFA for residual limb neuroma-associated pain during the period 2015 – 2019 were retrospectively reviewed. Information obtained included:

1. Patient demographics
2. Site of amputation
3. Reason for amputation
4. Size of neuroma and nerve involved
5. Phantom pain (if any)
6. RFA duration, temperature and pulsation (i.e. continuous versus alternating)
7. Pain (Numerical Rating Scale – NRS) scores – at 3-8 months pre-RFA, immediately pre and post RFA, 1 day, 2 days, 2 weeks and 3 months post-RFA.
8. Analgesic requirements pre- and post-RFA
9. Adverse effects of RFA (if any)
10. Comfort and ease of using prosthetic limb pre- and post-RFA.

After obtained the NRS pain scores at the aforementioned intervals, statistical analysis was performed using MATLAB (MathWorks, MA, USA). In order to assess the expected value and variation in pain scores amongst the patients, the mean pain score and standard deviation at the different time intervals (pre- and post-treatment) for all nine patients and for the six successfully treated patients were calculated.

In order to determine the statistical significance of the change in pain scores, Wilcoxon signed-rank test was used to calculate the p-values when examining the change in pain scores from initial presentation and the different time intervals (pre- and post-treatment). Wilcoxon signed-rank test was selected because of the small sample size and that pain scores cannot be assumed to be normally distributed.

Written informed consent was obtained from each patient prior to the RFA procedure. As this is a retrospective review of past patient notes, ethical approval was not needed.

### Radiofrequency Ablation Assessment and Protocol

**Figure 1:**
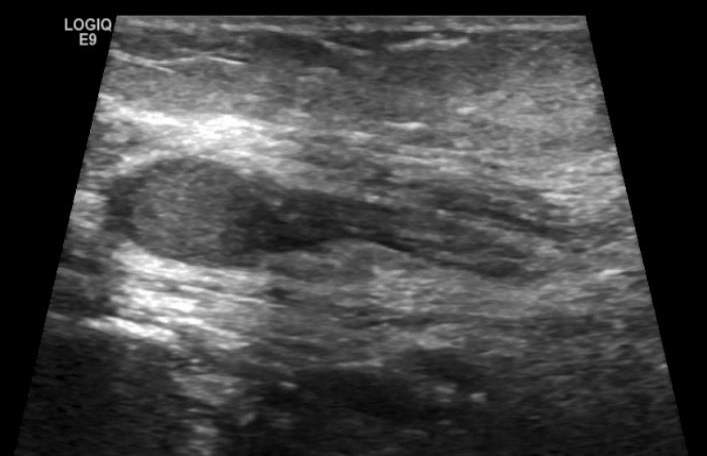
US image of 11x6mm neuroma along the ulnar nerve in the residual limb of a patient who had a trans-radial amputation (patient 1).

**Figure 2:**
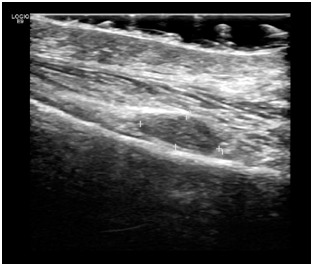
US image of 10x7 mm neuroma along the common peroneal nerve in the residual limb of a patient who had a transtibial amputation (patient 2).

**Figure 3:**
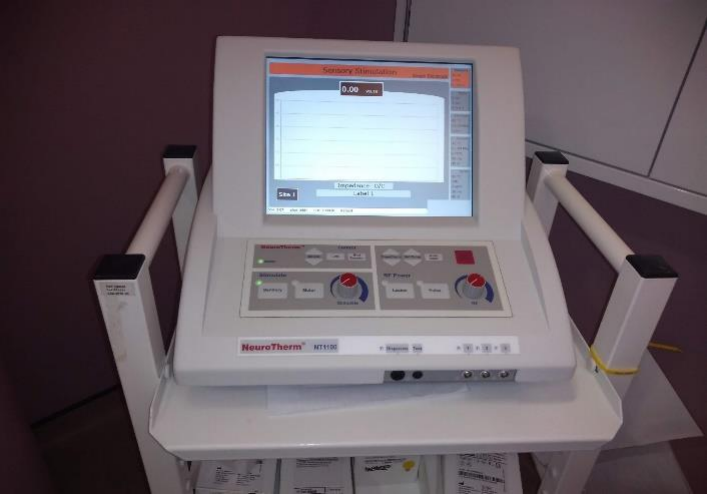
NeuroTherm NT1000 Radiofrequency Generator

**Figure 4:**
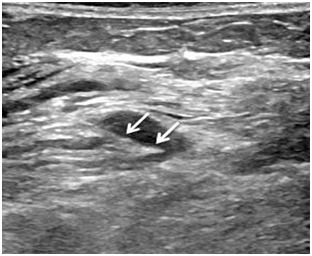
Advancing the electrode-cannula (arrowed) into the neuroma for RFA under US guidance

1. The initial assessment in the outpatient clinic of the patients with limb amputation was performed by a Consultant Rehabilitation Physician (DHS) or a senior trainee under the supervision of the consultant at which initial pain scores were taken.
2. Through magnetic resonance imaging (MRI), the presence a neuroma was confirmed as well as its site, size and nerve involved.
3. The patients were then reviewed again post-MRI scanning by the Consultant Rehabilitation Physician and referred to a single Consultant Musculoskeletal Radiologist (RM) who performed US-guided RFA treatment.
4. The patients waited between 3-8 months for their US-guided RFA treatment.
5. On the day of treatment, pain scores were taken again before the RFA.
6. The patient was placed in a comfortable position (supine for the lower limb, sitting for the upper limb). The skin was then prepped with Isopropyl Alcohol solution and draped.
7. The linear high-frequency ultrasonic transducer (5-13MHz; VFX13-5, Siemens AG) was covered with a sterile transparent sticker and placed transversely over the area of focal tenderness as reported by the patient.
8. On imaging with a high-resolution sonography machine (Acuson Antares; Siemens AG, Munich, Germany), the presence of the neuroma was reconfirmed on both transverse and longitudinal views by rotating the transducer by 90 degrees. The neuroma was visualised as a well-defined hypoechoic lesion involving the affected transected nerve (Figure 1, Figure 2).
9. After attaching to a radiofrequency generator (NeuroTherm NT1000; Morgan Innovation and Technology Ltd, Hampshire, UK) (Figure 3), a 10 cm disposable radiofrequency electrode was placed into a compatible 10 cm radiofrequency straight cannula (St Jude Medical, Plymouth, MN, USA) with a 5 mm active tip. With the transducer placed vertically to give a longitudinal view of the neuroma, this tip was advanced towards the nerve just proximal to the neuroma with the aim of reproducing the pain and confirming the site. Under direct imaging, 40 mg triamcinolone acetonide mixed with 2 mL 0.25% bupivacaine was injected around the nerve just proximal to the neuroma. Relief of the pain a few minutes after administration of the steroid confirmed the target neuroma. If neuroma-associated pain was confirmed using this technique, the tip of the electrode-cannula would be advanced into the affected neuroma (Figure 4). RFA would then be carried out 90°Celsius continuously for 9 minutes.
10. After undergoing RFA, pain scores were taken immediately post-procedure, via telephone follow up 1 day, 2 days and 2 weeks post-RFA, and via face to face follow up in the rehabilitation clinic 3 months post-RFA. The patients were asked to give the maximum pain score that they felt on the day of assessment.

## RESULTS

### Pre-treatment data

There were 9 patients (3 men and 6 women) aged between 30 and 69 years (mean 45 years). Eight had lower limb amputations (5 had transtibial, 2 transfemoral, 1 hip disarticulation) and one had a trans-radial amputation. Four of these patients underwent amputation due to trauma, two due to peripheral vascular disease, two due to neoplasm and one due to infection. The patients underwent amputation between 1 and 26 years ago (mean 7 years).

On initial presentation to the outpatient rehabilitation clinic, seven patients reported a pain severity of 10/10 on the NRS score, one had a pain score of 9 and one had a pain score of 7. All nine patients reported pain around the residual limb, and six of these patients also complained of phantom pain in addition to their residual limb pain. The residual limb condition in all these patients was stable with no local inflammation or acute skin or soft tissue changes. All patients reported discomfort and difficulty wearing their prosthetic limb due to pain. Patient demographics, prosth-eses suspension and weight-bearing characteristics, pre-RFA pain and neuroma characteristics are illustrated in Table1. The duration of pain symptoms from onset to initial presentation in clinic ranged from three months to six months with a mean of four months. Apart from simple and/or neuropathic analgesics, the patients had not received any other conservative pain-relieving interven-tions.

**Table 1:** Patient demographics, pre-RFA pain and neuroma characteristics.

Patient	Sex	Age	Amputation site	Cause of amputation	Years since amputation	Prosthesis suspension mechanism	Prosthesis socket weight bearing characteristics	Pain score (NRS) at presentation	Additional phantom pain at presentation	Pain causes discomfort/difficulty wearing prosthesis	Size of neuroma (mm) on MRI	Nerve involved
1	F	67	Transradial	Peripheral vascular disease	6	Suction	Non-weight bearing	10	+	Yes	11x6	Ulnar nerve
2	M	30	Transtibial	Trauma	2	Sleeve	Patellar Tendon Bearing	7	+	Yes	10x7	Common Peroneal nerve
3	M	27	Transtibial	Trauma	7	Elevated vacuum	Total surface bearing	10	-	Yes	14x8	Tibial nerve
4	F	37	Transtibial	Trauma	10	Pin lock (Clutch)	Total surfacing bearing	10	-	Yes	10x6	Tibial nerve
5	M	47	Hip disarticulation	Neoplasm	26	Strap	Ischial weight bearing	10	+	Yes	30x30	Sciatic nerve
6	F	49	Transtibial	Peripheral vascular disease	6	Sleeve	Patellar Tendon Bearing	9	+	Yes	12x10	Tibial nerve
7	F	42	Transfemoral	Neoplasm	1	Belt	Ischial weight bearing	10	-	Yes	18x13	Sciatic nerve
8	F	69	Transtibial	Infection	5	Sleeve	Patellar Tendon Bearing	10	+	Yes	12x12	Superficial peroneal nerve
9	F	36	Transfemoral	Trauma	3	Lanyard	Ischial weight bearing	10	+	Yes	19x15	Sciatic nerve

**Figure 5:**
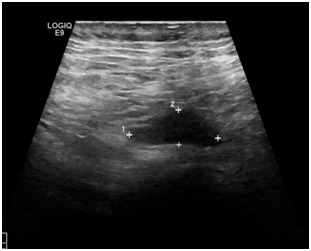
US image of 30x30 mm neuroma along the sciatic nerve in a patient who had a hip disarticulation (patient 5).

Through magnetic resonance imaging (MRI) and ultrasound scanning, three patients were found to have neuromas involving the tibial nerve, three involved the sciatic nerve, one involved the common peroneal nerve, one the superficial peroneal nerve and one the ulnar nerve. The largest neuroma was 30x30mm and involved the sciatic nerve (Figure 5). The smallest neuroma was 10x6mm and involved the tibial nerve

The nine patients were consecutively referred from the prosthetic rehabilitation team to the radiology team for consideration for ultrasound-guided RFA. On the day of treatment, pain scores were taken again before the RFA – six patients reported a score 10/10 for their pain, two patients reported a score 8/10 and one patient reported a score 7/10.

In all cases, the patient was able to point to a focal area of tenderness over the residual limb that was elicited on direct palpation. In the six patients with phantom pain, palpating over the focal area of tenderness led to a positive Tinel’s sign along the course of the affected nerve in the phantom limb. On direct pressure from the transducer, the pain reported by the patient in all cases was able to be reproduced.

### Post-treatment results

After undergoing RFA, pain scores were taken immediately post-procedure, via telephone follow up 1 day, 2 days and 2 weeks post-RFA, and via face to face follow up in the rehabilitation clinic 3 months post-RFA (Table 2).

**Table 2:** Results of RFA treatment in terms of changes in pain, analgesic requirements and ease/comfort of prosthesis use.

Patient	Pain score (NRS) immediately pre-RFA	Pain score (NRS) immediately post-RFA	Pain score (NRS) Day 1 post-RFA	Pain score (NRS) Day 2 post-RFA	Pain score (NRS) 2 weeks post-RFA	Pain Score (NRS) at 3 months post- RFA	Phantom pain post-RFA	Changes in Analgesic requirements pre-and post RFA	Improved ease and comfort of wearing prosthesis post-RFA	Adverse effects / other issues
1	10	0	0	0	10	10	+	No change (still on gabapentin 300mg TDS)	No	Did not have diagnostic steroid injection pre-RFA
2	8	7	6	5	0	0	-	Gabapentin reduced from 600mg TDS to 300mg TDS	Yes	None
3	7	2	0	0	0	2	-	Did not take analgesics pre- or post RFA	Yes	No
4	10	0	0	0	0	0	-	Did not take analgesics pre- or post RFA	Yes	No
5	10	9	8	7	2	10	+	Did not take analgesics pre- or post RFA	No	Required surgical excision of neuroma
6	10	9	8	1	0	1	-	Gabapentin reduced from 600mg TDS to 300mg TDS	Yes	None
7	10	7	7	7	10	6	-	No longer taking oxycodone 5mg BD	No	Adjacent bony spur
8	8	8	8	8	6	3	-	Paracetamol1g QDS moved to PRN	Yes	None
9	10	5	1	1	unknown	0	+	No change (still on pregabalin 75mg ON, amitriptyline 25mg ON)	Yes	None

All patients bar one underwent a diagnostic US-guided steroid injection to confirm that the neuroma was the source of the pain, prior to RFA.

Six patients reported a significant reduction in pain NRS scores – defined as at least 50% reduction,7 sustained over at least 3 months with no adverse effects. Three of these six patients also reported a reduction in analgesic requirements, two patients did not have any analgesic pre- and post-RFA and one patient did not report any significant change in analgesic demands. All of the six patients reported an improvement in comfort in wearing their prosthetic limb. Amongst these six patients who reported significant pain relief, three of them also had pre-existing phantom pain and found that RFA also resolved their phantom pain completely.

Of the three other patients who did not report a significant sustained reduction overall pain, one had a large sciatic nerve neuroma (Figure 5) which was eventually successfully treated with surgical excision.

Another patient was found to have a bony spur adjacent to the neuroma at the time of the ultrasound-guided RFA which could have contributed to her pain, whilst the third patient reported an initial reduction in pain for 1-week post RFA but then experienced a return of the pain including heightened phantom sensation. Interestingly, the third patient did not receive a routine diagnostic steroid injection prior to undertaking the RFA as we had confidently, but erroneously, assumed that the large neuroma was the cause of her pain. Apart from failure to relieve the pain in three of the patients, no complications of RFA were reported in all nine patients and no pathological changes were observed at the distal end of the targeted nerve during and after RFA.

Routine regular follow up was carried out up the three-month mark post-RFA, after which the patients were given the option to return to the clinic through a self-referral (via an open appointment) or re-referral via their general practitioner/family physician if there were any further problems with pain or discomfort. At the time of writing, none of the six successfully treated patients chose to return to clinic and thus we have not observed any reports of recurrence in the longer term.

### Statistical Analysis

Statistical analysis was performed using MATLAB (MathWorks, MA, USA). The mean pain score and standard deviation at the different time intervals (pre- and post-treatment) for all nine patients and for the six successfully treated patients were calculated. This data is tabulated in Table 3 (A and B) and graphically illustrated in Appendix.

**Table 3:** Mean pain score, standard deviation (SD) and *p*-values at different time intervals pre- and post-RFA of all nine patients (A) and six successfully treated patients (B).

		A			B	
Time interval	Mean	SD	*p*-value	Mean	SD	*p*-value
At presentation	9.56	1.01	1.0000	9.33	1.21	1.0000
Immediately pre-RFA	9.22	1.20	0.6250	8.83	1.32	0.6250
Immediately post-RFA	5.22	3.67	0.0156	5.17	3.55	0.1250
Day 1	4.22	3.83	0.0039	3.83	3.92	0.0313
Day 2	3.22	3.46	0.0039	2.50	3.27	0.0313
2 weeks	3.50	4.21	0.0156	1.58	2.58	0.0313
3 months	3.56	4.13	0.0156	1.00	1.26	0.0313

Wilcoxon signed-rank test calculated the p-values of the changes in pain scores at initial presentation and the different time intervals pre- and post-treatment (immediately before treatment, immediately after treatment, 1 day, 2 days, 2 weeks and 3 months post-treatment) for all nine patients and for the six successfully treated patients (Table 3, A, B). The p-values comparing the change in pain scores at initial presentation and from Day 1 post-treatment were consistently below 0.05, which is strongly against the null hypothesis and suggests a statistically significant reduction in pain scores.8 Interestingly, the *p*-values for all nine patients were lower than the p-values for the six successfully treated patients. This could be explained by two factors. Firstly, Patient 1 reported an initial complete resolution of her pain (from 10 to 0 on the NRS) followed by a full return of her pain two weeks later. This outlier may have unduly influenced the p-value calculations. Secondly, reducing the already small sample size from nine patients to an even smaller sample of six patients could also have increased the *p*-values.

## DISCUSSION

Neuroma formation is common after limb amputation. The type commonly found post-amputation are terminal neuromas which can occur when the proximal nerve terminal is severed during the surgery, after which apoptosis occurs in the distal axons of the nerve.^9^ In order to maintain the congruity of the axon, Schwann cells stimulate new growth of adjacent axons. However, in cases where the defect of the axon is too long to overcome by the aforementioned mechanism, the proximal axons will growth in multiple directions to overcome the defect, giving a bulbous appearance to the neuroma that is visualised on MRI and ultrasound imaging.^9^

Residual limb neuroma-associated pain can be difficult to treat. A multidisciplinary approach is often required in terms of adjustments to the liner and socket by the prosthetists, psychological input, gait retraining by physiotherapy, oral analgesic changes, specialist input by the pain team and ultimately surgical excision with its associated operative risks and risk of recurrence.^10^

There is a paucity of studies in the literature concerning ultrasound-guided RFA in the treatment of residual limb neuroma-associated pain in individuals with limb amputation. These have mainly been isolated case reports.^10-12^ There has been one previous case series carried out by Zhang et al of 13 patients exploring the use of alcohol neurolysis in combination with RFA in residual limb neuroma associated pain.^13^ The authors found that in patients whose neuroma pain was resistant to alcohol neurolysis, RFA provided an effective alternative in terms of pain relief. RFA may be continuous or pulsed. Continuous RFA uses high-frequency alternating current which leads to coagulative necrosis of the neuroma. Pulsed RFA utilises the current in short 20 millisecond bursts with a half-second respite in between to allow for heat dissipation.^14^

In these isolated case reports, pulsed RFA was performed at 42°C for 120-240 seconds each in two to three separate intervals, whereas in Zhang et al case series pulsed RFA was carried out at 80°C for 240 seconds each in two separate intervals. and the authors suggested this gave more consistent favourable results in terms of longer-term improvements in pain scores up to 6 months post-procedure.

There is currently no consensus on the temperature and duration for RFA or whether it should be continuous or pulsed. Our study differs from the existing literature in that we have used continuous RFA at 90°C for nine minutes (540 seconds) which is in line with our local hospital practice. In addition to the higher temperature and longer duration, we have found that a single intervention of continuous RFA was sufficient in most of our patients in terms of sustained long-term pain relief without the need for multiple RFA treatments as seen Zhang et al’s study involving pulsed RFA. Continuous RFA is known to be more effective than pulsed RFA in the treatment of facet joint-related low back pain ^14-16^ and a similar trend may also be apparent in residual limb neuroma associated pain in individuals with limb amputation.

In the six patients who reported significant pain relief post-RFA, three of them also had pre-existing phantom pain and found that RFA also resolved their phantom pain completely. There are many theories as to where phantom pain originates including peripheral, spinal and central nervous system contributors. Our study supports the theories that there is a peripheral contribution. This is also supported by as Zhang et al who note that in clinical practice residual limb pain and phantom pain are often intertwined and can be difficult to separate. Severing of peripheral nerves during amputation can lead to hyperexcitability and spontaneous generation of action potentials from the cut nerve, which in turn can cause phantom pain. This theory could explain the growing use of peripheral nerve blocks to treat phantom limb pain.^17-19^

Undergoing a diagnostic steroid injection prior to the RFA may be a confounding factor in terms of the patient’s pain relief. US-guided steroid injections have been used to treat painful residual limb neuroma with promising early results in a few isolated case reports and a small case series.^20-22^ But again, like with RFA and residual limb neuromas, the number of studies and the number of patients treated could be too small to infer any concrete consensus.

**Figure 6:**
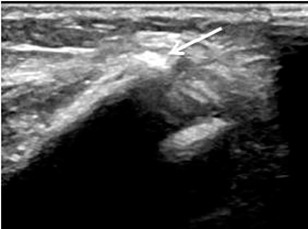
US image of a bony spur (arrowed) adjacent to a 18x13mm neuroma along the sciatic nerve in a patient who had a transfemoral amputation (patient 7).

As mentioned, of the three other patients who did not report a significant reduction in pain, one had a large sciatic nerve neuroma that was eventually successfully treated with surgical excision, one had confounding pain from an adjacent bony spur (Figure 6) that was also eventually surgically excised, whilst the third patient did not receive a routine diagnostic steroid injection confirming the neuroma as the source of the pain prior to undertaking RFA.

This sciatic nerve neuroma (30 x 30 mm) was almost double the size of the second-largest neuroma in our study. This might suggest that RFA would be better suited to treating pain originating from small to medium size neuromas.

For larger neuromas, surgical intervention may be an inevitability. Dumanian et al carried out a randomised controlled trial of 28 individuals with limb amputation with painful residual limb neuroma assigned to either traditional surgical excision or Target Muscle Reinnervation (TMR).^23^ During surgical excision of a neuroma, TMR involves identifying using a nerve stimulator nearby sensory and motor nerves innervating surrounding muscle and suturing the nerves to a surgically divided distal nerve (e.g. tibial nerve sutured to the distal segment of the motor nerve to the soleus). This nerve transfer technique aims to facilitate reinnervation as close as possible to resembling physiological innervation thus potentially inhibiting the pathological central reorganisation of neuropathic pain mechanisms.^24^ The authors found that TMR provided greater pain relief for both residual limb pain and phantom pain on the NRS compared with traditional surgical excision of the neuroma.^23^ The same research group also conducted a multicentre cohort study comparing 51 patients undergoing major limb amputation with immediate TMR with 438 unselected standard major limb amputation patients. The authors found that immediate TMR at amputation reduced both phantom and residual limb pain on the NRS and may also reduce the formation of neuromas in the first place.^24^

Traditional surgical excision has also been criticised in terms of the invasiveness of the dissection. A large and invasive dissection can lead to delayed wound healing as well as excessive adhesions and scar tissue formation, which can cause pain and discomfort during prosthesis fitting. This, in turn, can lead to delays in weight-bearing and rehabilitation, A large and invasive dissection of the neuroma can also be associated with higher recurrence rates.^25^ It is for these reasons that less invasive surgical excision techniques have been explored. Thomas et al conducted a case series of 10 patients with limb amputation who underwent ultrasound-guided needle localisation of their painful neuroma prior to surgical excision. The authors found that pre-operative US-guided localisation of the neuroma facilitate less invasive surgical dissections and thus potentially a reduction in the aforementioned complications.^25^

Limitations to our study include the relatively small sample size and lack of a control group, and thus prospective cohort studies with a larger sample size may be needed to confirm our results before it can be generalised to the larger population of individuals with limb amputation with residual limb neuroma.

## CONCLUSION

Our retrospective case series suggests that US-guided continuous RFA is a safe and effective treatment for residual limb neuroma associated pain and phantom pain in individuals with limb amputation. US-guided RFA can reduce pain and analgesic requirements, improve comfort and ease of wearing the prosthesis and reduce the potential need for surgical excision of the neuroma and its associated surgical risks.

We recommend all patients undergo a diagnostic steroid injection prior to RFA to confirm that the residual limb neuroma is the source of the pain.

Further studies are needed to study the co-factors that could determine the residual limb pain and could affect the RFA treatment. This could facilitate a pre-selection of the responders to the treatment. We plan on conducting a prospective longitudinal series study with a larger sample size to investigate further the effect of RFA treatment of neuroma-associated residual limb and phantom pain in individuals with limb amputation including comparing different temperatures, durations, continuous versus pulsed RFA, as well as exploring combined RFA and phenol / alcohol injections (neurolysis).

## DECLARATION OF CONFLICTING INTERESTS

The authors declare no conflict of interest.

## SOURCES OF SUPPORT

The authors received no financial or other sources of support for this study.

## AUTHOR CONTRIBUTION

**Dr Henderson Slater** and **Dr Mansour** initially thought of the study. All three authors devised the study design. Dr Mansour devised the local protocol for RFA practice, collected pain scores up to 2 weeks post-treatment and the sonographic images. **Dr Guo** retrieved the clinical data from the patient notes, analysed and interpreted the data, and performed the statistical analysis. Dr Guo wrote and revised the manuscript with advice from Dr Henderson Slater.

## APPENDIX

**Figure:**
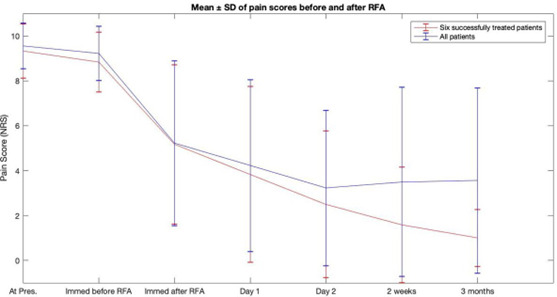
Mean ± Standard deviation (SD) of pain scores at different time intervals pre- and post-RFA

## References

[1] Gruber H, Kovacs P, Peer S, Frischhut B, Bodner G. Sonographically Guided Phenol Injection in Painful. AJR Am J Roentgenol. 2004;182:952–4. DOI: 10.2214/ajr.182.4.1820952 15039170

[2] Mathews GJ, Osterholm JL. Painful traumatic neuromas . Surg Clin North Am. 1972;52(5):1313–28. DOI:10.1016/s0039-6109(16)39843-7 4561764

[3] Restrepo-Garces CE, Marinov A, McHardy P, Faclier G, Avila A. A.Pulsed radiofrequency under ultrasound guidance for persistentstump-neuroma pain. Pain Pract. 2011;11(1):98–102. 10.1111/j.1533-2500.2010.00398.x 20642489

[4] Hsu E, Cohen SP. Postamputation pain: epidemiology mechanisms, and treatment. J Pain Res. 2013;6–121. doi:10.2147/JPR.S32299 PMC3576040 23426608

[5] Ehde DM, Czemiecki JM, Smith DG, Campbell KM, Edwards WT, Jensen MP, et al. Chronic phantom sensations, phantom pain, residual limb pain, and other regional pain after lower limb amputation. Arch Phys Med Rehabil. 2000;81(8):1039–44. 10.1053/apmr.2000.7583 10943752

[6] Montoya P, Larbig W, Grulke N, Flor H, Taub E, Birbaumer N. The relationship of phantom limb pain to other phantom limb phenomena in upper extremity amputees. Pain. 1997;72(1-2):87–93. 10.1016/S0304-3959(97)00004-3 9272791

[7] Bendinger T, Plunkett N. Measurement in pain medicine. BJA Educ. 2016 Sep 1;16(9):310–5. https://doi.org.proxy.bib.uottawa.ca/10.1093/bjaed/mkw014

[8] Perneger TV., Combescure C . The distribution of P-values in medical research articles suggested selective reporting associated with statistical significance. J Clin Epidemiol. 2017 1;87:70–7. 10.1016/j.jclinepi.2017.04.003 28400294

[9] Kang J, Yang P, Zang Q, He X. Traumatic neuroma of the superficial peroneal nerve in a patient: A case report and review of the literature. World J Surg Oncol. 2016; 14(1). DOI:10.1186/s12957-016-0990-6 PMC5018173 27613606

[10] Restrepo-Garces CE, Marinov A, McHardy P, Faclier G, Avila A. Pulsed Radiofrequency Under Ultrasound Guidance for Persistent Stump-Neuroma Pain. Pain Pract. 2011;11(1):98–102. 10.1111/j.1533-2500.2010.00398.x 20642489

[11] Kim YK, Jung I, Lee CH, Kim SH, Kim JS, Yoo and BW. Pulsed Radiofrequency Ablation Under Ultrasound Guidance for Huge Neuroma. Korean J Pain. 2014;27(3):290–3. 10.3344/kjp.2014.27.3.290 25031817 PMC4099244

[12] Zheng B, Song L, Liu H. Pulsed radiofrequency of brachial plexus under ultrasound guidance for refractory stump pain: A case report. J Pain Res.. 2017 7;10:2601–4. doi:10.2147/JPR.S148479 29158692 PMC5683784

[13] Zhang X, Xu Y, Zhou J, Pu S, Lv Y, Chen Y, et al. Ultrasound-guided alcohol neurolysis and radiofrequency ablation of painful stump neuroma: Effective treatments for post-amputation pain. J Pain Res. 2017;10:295–302. doi: 10.2147/JPR.S127157 28223839 PMC5305268

[14] Jena B, Paswan A, Singh Y, Loha S, Singh A, Rastogi V. A comparative study of continuous versus pulsed radiofrequency discectomy for management of low backache: Prospective randomized, double-blind study. Anesth Essays Res. 2016;10(3):602. doi: 10.4103/0259-1162.186616 27746559 PMC5062218

[15] Lopez WO, Navarro PA, Vargas MD, Alape E, Lopez PA. Pulsed radiofrequency versus continuous radiofrequency for facet joint low back pain: a systematic review. World neurosurgery. 2018. 390–6. 10.1016/j.wneu.2018.10.191 30404055

[16] Pangarkar S, Miedema ML. Pulsed Versus Conventional Radio Frequency Ablation for Lumbar Facet Joint Dysfunction. Curr Phys Med Rehabil Reports. 2014;2(1):61–5. DOI 10.1007/s40141-013-0040-z

[17] llfeld BM, Moeller-Bertram T, Hanling SR, Tokarz K, Mariano ER, Loland VJ, et al. Treating intractable phantom limb pain with ambulatory continuous peripheral nerve blocks: A pilot study. Pain Med (United States). 2013;14(6):935–42. https://doi-org.proxy.bib.uottawa.ca/10.1111/pme.12080 10.1111/pme.12080 PMC3696484 23489466

[18] Gilmore C, llfeld B, Rosenow J, Li S, Desai M Hunter C, et al. Percutaneous peripheral nerve stimulation for the treatment of chronic neuropathic postamputation pain: A multicenter, randomized, placebo-controlled trial. Reg Anesth Pain Med. 2019;44(6):637–45. 10.1136/rapm-2018-100109 30954936

[19] Borghi B, D'Addabbo M, Borghi R. Can neural blocks prevent phantom limb pain? Pain Manag. 201;4(4):261–6. 10.2217/pmt.14.17 25300383

[20] Chen P-J, Liang H-W, Chang K-V, Wang T-G. Ultrasound-guided injection of steroid in multiple postamputation neuromas. J Clin Ultrasound. 2013;41(2):122–4. 10.1002/jcu.21885 22290559

[21] Hung YH, Wu CH, Özçakar L, Wang TG. Ultrasound-guided steroid injections for two painful neuromas in the stump of a below-elbow amputee. Am J Phys Med Rehabil. 2016;95(5):e73. DOI:10.1097/PHM.0000000000000456 26945215

[22] Kesikburun S, Yasar E, Dede I, Göktepe S, Tan AK. Ultrasound-guided steroid injection in the treatment of stump neuroma: pilot study. J Back Musculoskelet Rehabil. 2014;27(3):275–9. DOI: 10.3233/BMR-130444 24284273

[23] Dumanian GA, Potter BK, Mioton LM, Ko JM, et al. Targeted Muscle Reinnervation Treats Neuroma and Phantom Pain in Major Limb Amputees: A Randomized Clinical Trial. Ann Surg. 2019;270(2):238–46. doi:10.1097/SLA.0000000000003088 30371518

[24] Valerio IL, Dumanian GA, Jordan SW, Mioton LM, Bowen JB, West JM, et al. Preemptive Treatment of Phantom and Residual Limb Pain with Targeted Muscle Reinnervation at the Time of Major Limb Amputation. J Am Coll Surg. 2019;228(3):217–26. 10.1016/j.jamcollsurg.2018.12.015 30634038

[25] Thomas AJ, Bull MJ, Howard AC, Saleh M. Peri operative ultrasound guided needle localisation of amputation stump neuroma. Injury. 1999;30(10):689–91. 10.1016/S0020-1383(99)00185-0 10707244

